# Characterization of the Biocontrol Activity of *Pseudomonas fluorescens* Strain *X* Reveals Novel Genes Regulated by Glucose

**DOI:** 10.1371/journal.pone.0061808

**Published:** 2013-04-15

**Authors:** Gerasimos F. Kremmydas, Anastasia P. Tampakaki, Dimitrios G. Georgakopoulos

**Affiliations:** Department of Agricultural Biotechnology, Agricultural University of Athens, Athens, Greece; University of the West of England, United Kingdom

## Abstract

*Pseudomonas fluorescens* strain X, a bacterial isolate from the rhizosphere of bean seedlings, has the ability to suppress damping-off caused by the oomycete *Pythium ultimum*. To determine the genes controlling the biocontrol activity of strain X, transposon mutagenesis, sequencing and complementation was performed. Results indicate that, biocontrol ability of this isolate is attributed to *gcd* gene encoding glucose dehydrogenase, genes encoding its co-enzyme pyrroloquinoline quinone (PQQ), and two genes (*sup*5 and *sup*6) which seem to be organized in a putative operon. This operon (named *sup*X) consists of five genes, one of which encodes a non-ribosomal peptide synthase. A unique binding site for a GntR-type transcriptional factor is localized upstream of the *sup*X putative operon. Synteny comparison of the genes in *sup*X revealed that they are common in the genus *Pseudomonas*, but with a low degree of similarity. *sup*X shows high similarity only to the mangotoxin operon of *Ps. syringae* pv. *syringae* UMAF0158. Quantitative real-time PCR analysis indicated that transcription of *sup*X is strongly reduced in the *gcd* and PQQ-minus mutants of *Ps. fluorescens* strain X. On the contrary, transcription of *sup*X in the wild type is enhanced by glucose and transcription levels that appear to be higher during the stationary phase. Gcd, which uses PQQ as a cofactor, catalyses the oxidation of glucose to gluconic acid, which controls the activity of the GntR family of transcriptional factors. The genes in the *sup*X putative operon have not been implicated before in the biocontrol of plant pathogens by pseudomonads. They are involved in the biosynthesis of an antimicrobial compound by *Ps. fluorescens* strain X and their transcription is controlled by glucose, possibly through the activity of a GntR-type transcriptional factor binding upstream of this putative operon.

## Introduction

Many bacterial strains from the genus *Pseudomonas* are capable of suppressing a range of plant diseases caused by soil-borne plant pathogenic fungi, due to their ability to biosynthesize antimicrobial metabolites. Antibiotics, cyclic lipopeptides (CLPs) with antimicrobial activity, siderophores and hydrogen cyanide are the main secondary metabolites to which the biological control is attributed [1]. Regulation of the biosynthesis of these antimicrobial metabolites has been extensively studied. A wide range of environmental as well as endogenous factors control the transcription of several genes involved in the biosynthesis of antimicrobial metabolites [2], [3].

Glucose is one of the environmental factors which affect the biosynthesis of secondary metabolites such as oomycin A [4], kanosamine [5], DAPG [6], pyoluteorin and pyochelin [7], prodigiosin [8], pyrrolnitrin and phenazine [9]. Recently, it has been proposed that it is gluconic acid, not glucose, that regulates the production of antimicrobial metabolites [10]. Moreover, gluconic acid has been suggested as having a direct inhibitory effect on phytopathogenic fungi sensitive to lower concentrations of the acid [11].

Gluconic acid derives from glucose by an oxidative reaction in the periplasmic space, thus affecting the pH and the availability of soluble phosphates in all glucose-containing media [12]. The oxidation of glucose to gluconic acid is catalysed by membrane-bound quinoprotein glucose dehydrogenases (Gcd) that are involved either in biocontrol of plant pathogens [10], [13] or in pathogenicity of bacteria in mammals [14]. Among various quinoprotein dehydrogenase enzymes in bacteria, Gcd uses pyrroloquinoline quinone (PQQ) as an essential cofactor [15]. Genes involved in the biosynthesis of PQQ are organised in a putative gene cluster that is expressed as an operon and its transcription is regulated by various carbon sources [16]. Insertional inactivation of the PQQ biosynthetic genes has proven their significance in biocontrol [17], [18], [19], [13] and in plant growth promotion [20].

A rise of interest in CLP metabolites has been recently noted, due to their biosurfactant, antimicrobial and phytototoxic activity [21]. The biosynthetic model of various CLPs has been fully elucidated, yet regulation for most of them is still under study [22]. Environmental factors such as pH, temperature, carbon and nitrogen sources [23], [24], plant signal molecules [25] and protozoan predators [26] affect the production of CLP metabolites. Also, endogenous factors like the two component regulatory system GacA/GacS [27], [28], [29], quorum sensing [30], [31], [32], [33], sigma factors and Hsp [34] control the expression of several CLP biosynthetic genes.

*Pseudomonas fluorescens* strain X is a bacterial biocontrol agent able to suppress cucumber and sugar beet damping-off caused by *Pythium ultimum*. Suppression of damping-off by *Ps. fluorescens* strain X has been proved to be more effective over other *Pseudomonas* and *Bacillus* strains [35]. Nevertheless, its biocontrol ability has not yet, been linked to any known antimicrobial metabolites.

The aim of the present study was to elucidate the mechanism by which *Ps. fluorescens* strain X suppresses damping-off. In order to achieve this, transposon mutants of strain X were created (designated sup^−^) which were impaired in their ability to suppress the *in vitro* radial growth of *P. ultimum*. Sup^−^ mutants were found to carry the transposon integration in six different genes. Results indicate that genes *sup*5 and *sup*6 have key role in the biosynthesis of a antimicrobial metabolite, while genes *sup*1, *sup*2, *sup*3 and *sup*4 play a secondary role, indirectly controlling the transcription of the first two.

## Results

### Biochemical Characterization of *Ps. fluorescens* Strain X and Derived Mutants

Nine mutants (k36, W139, R48, B161, B91, A150, ρ26, δ40 and ρ93) were isolated out of a mutant library of 12000 derived from random Tn5 insertion mutagenesis of *Ps. fluorescens* strain X. All mutants had lost the ability to inhibit *P. ultimum* radial growth on Potato Dextrose Agar (PDA) and retained the same growth rate with the wild type (data not shown). When the wild type was incubated on minimal medium (M9) supplemented with glucose (2% w/v) as the sole carbon source, it acidified the substrate, lowering the pH from 6 to 5. On the contrary, mutants k36, W139, R48, B139, B91, A150 and ρ26 increased the pH of the medium from 6 to 8, while δ40 and ρ93 did not alter the pH (Table 1). Furthermore, cell-free filtrates of the wild type strain and the complemented mutants (described in a subsequent section) after growth in either Potato Dextrose Broth (PDB) or Luria Bertani Broth (LB), were treated overnight with proteinase K and pronase and were tested for inhibition of *P. ultimum* radial growth (Table 1). No alterations in the antimicrobial activity for any of the treated filtrates were observed (Table 1). These results indicate that the antimicrobial activity might be attributed to a molecule either of non-peptidic nature or containing non-proteinaceous aminoacids.

**Table 1:** Characteristics of sup^−^ mutants and complementation analysis.

Strain	Acidification^a^	*In vitro* inhibition of*P. ultimum* radial growthby PDB filtratesof bacteria	*In vitro* inhibition of*P. ultimum* radialgrowth by LB filtratesof bacteria	Antimicrobial activity of PDB filtrates after treatment with proteinase K and pronase^b^
X	+	+	−	+
k36/pBBRgcd1	+	+	−	+
k36	−	−	−	−
W139/pBBRgcd1	+	+	−	+
W139	−	−	−	−
R48/pBBRgcd1	+	+	−	+
R48	−	−	−	−
B163/pBBRpqqF-E	+	+	−	+
B163	−	−	−	−
B91/pBBRpqqF-E	+	+	−	+
B91	−	−	−	−
A150/pBBRpqqF-E	+	+	−	+
A150	−	−	−	−
ρ26/pBBRpqqF-E	+	+	−	+
ρ26	−	−	−	−
δ40/pBBRsupD	ND	+	−	+
δ40	ND	−	−	−
ρ93/pBBRsupD	ND	+	−	+
ρ93	ND	−	−	−

ND: Not Detected.

a Acidification was observed on solid minimal medium M9 supplemented with 2% w/v glucose, as described before [14].

b Enzyme treatment was performed for filtrates from incubation in PDB, as described before [47].

### Cloning and Sequencing of the Suppression Genes

**Figure 1:**
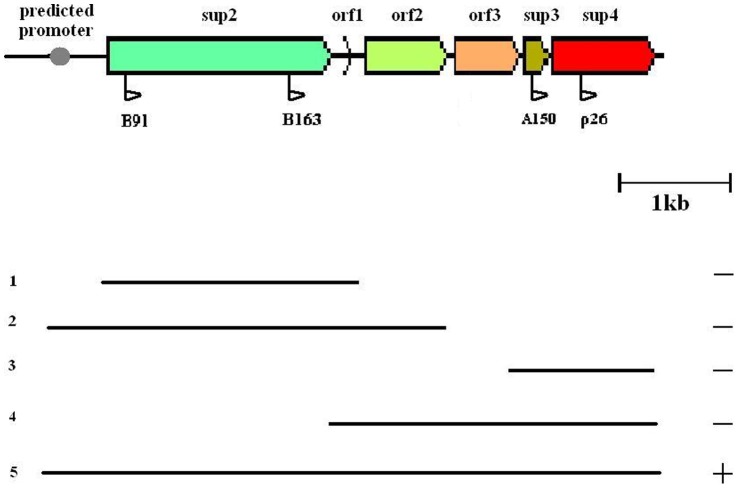
Complementation analysis of the PQQ biosynthesis region in *Ps. fluorescens* strain X. PCR fragments of this region (1–5), with different sets of genes from *Ps. fluorescens* X, and their ability to complement sup^−^ mutants. Ability to complement is noted with plus (+) or minus (−). The direction of the plasposon Tn*5*-RL27 insertion in the derivative mutants B91, B163, A150 and ρ26 is indicated with a flag beneath the sequence. The predicted site for the unique putative promoter is also marked.

**Figure 2:**
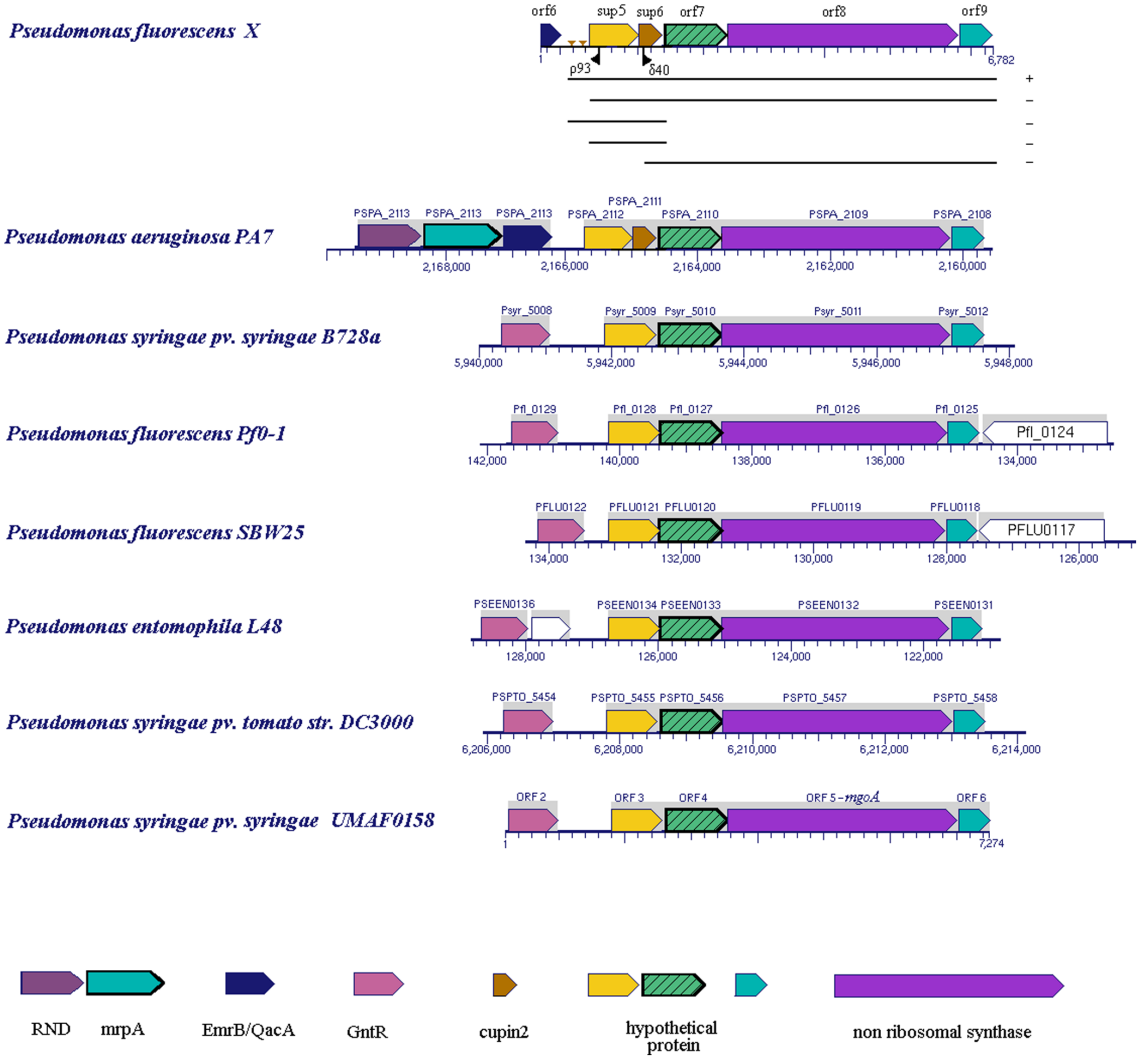
Arrangement of the genes in the genomic locus of *sup*5 and *sup*6, compared to other *Pseudomonas* strains, and complementation analysis of the region. The lines beneath the genomic of *Ps. fluorescens* X represent regions of this locus that were PCR-amplified, cloned into pBBR1MCS5 and tested for complementation. Ability to complement is noted with plus (+) or minus (−). Putative ORFs are indicated by thick coloured arrows on a line. Genes that might be organised in a putative operon are enclosed by a grey frame. The direction of the plasposon Tn*5*-RL27 insertion in mutants δ40 and ρ93 is indicated with a black arrow beneath the sequence (▸). Predicted sites for the unique putative promoter and operator are also marked (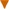;). Size, genomic location and locus tag of the different ORFs sequenced in *Ps. aeruginosa* PA7, *Ps. fluorescens* Pf0–1, *Ps. fluorescens* SBW25, *Ps. entomophila* L48, *Ps. syringae* pv. *syringae* B728a,*Ps. syringae* pv. *tomato* DC3000 and *Ps. syringae* pv. *syringae* UMAF0158 are indicated.

Southern hybridization confirmed that each of the mutants used in this study contained a single transposon insertion in the chromosome. Mutants containing more than one insertion were not analysed further (data not shown). Subsequent cloning and sequencing of the chromosomal regions flanking the transposon insertion in the selected mutants revealed that the transposon was localized in three different genomic regions. In mutants k36, W139 and R48 the transposon integration was in a 2,418 bp gene, designated *sup*1. The transposon insertion in mutants B163, B91 was in *sup*2, (2,424 bp). In mutants A150 and ρ26 the transposon was in two genes, *sup*3 and *sup*4, (276 bp and 996 bp respectively). Genes *sup*2, *sup*3 and *sup*4 were neighbouring in a genomic region of 7119 bp (Fig. 1). Finally, mutants δ40 and ρ93 had a transposon integration in genes *sup5* (777 bp) and *sup6* (348 bp) respectively. These genes were localized in a third genomic region of 6,782 bp (Fig. 2).

### Identification and Characterization of the Chromosome Regions Containing the Suppression Genes

The deduced product of *sup*1 (805 amino acids; 85.9 kDa) exhibits the highest similarity (95% identical) to the putative PQQ-dependent glucose dehydrogenase (Gcd) of the fully sequenced biocontrol strain *Ps. fluorescens* SBW25 encoded by PFLU1086 (GenBank accession no. YP_002870745.1). The product of *sup*1 was 70% identical to the Gcd from the well-characterized strain *Ps. fluorescens* CHA0 (ACN53518.1), encoded by locus FJ694890 (10), as well as to the Gcd from the biocontrol strain *Ps. fluorescens* Pf-5, encoded by PFL_4916 (AAY94145.1).

The proteins encoded by the neighbouring genes *sup*2, *sup*3 and *sup*4, show high similarity to PqqF, PqqD and PqqE respectively, involved in the biosynthesis of pyrroloquinoline quinone (PQQ). Sup2 (807 amino acids; 87.9 kDa) exhibits relatively low similarity (61% identical) to the putative peptidase PqqF of *Ps. fluorescens* CHA0 (CAA60730.1) [17]. The putative product of *sup*3 (91 amino acids; 10.3 kDa) has the highest similarity (96%) with PqqD of *Ps. fluorescens* SBW25 (YP_002875096.1). The deduced product of *sup*4 (331 amino acids; 37.4 kDa) showed 99% identity to the PqqE of *Ps. fluorescens* SBW25 (YP_002875097.1). The genes *sup*5 and *sup*6 are located in a genomic region similar to the *Ps. syringae* pv.*syringae* UMAF0158 mangotoxin biosynthesis region. The deduced product of gene *sup*5 (258 amino acids; 28.7 kDa) is a protein of unknown function with highest similarity (73% identical) to the protein encoded by *orf*3 from *Ps. syringae* pv. *syringae* UMAF0158 (ABG00044.1). Interestingly, the deduced product of *sup*5 is less similar to the proteins encoded from other *Ps. fluorescens* strains. Sup5 is 69% similar to the protein encoded by Pfl01_0128 from *Ps. fluorescens* Pf0–1 (YP_345861.1) and 53% similar to the protein encoded by PFLU_0121 from *Ps. fluorescens* SBW25 (YP_002869817.1). Furthermore, *sup*6 encodes a protein (115 amino acids; 13.3 kDa) that belongs to the cupin superfamily. The cupin superfamily, whose name comes from a conserved β-barrel fold structure (deriving from the Latin term ‘*cupa*’ that stands for a small barrel), is a functionally diverse family that comprises enzymatic and non-enzymatic members [36]. Among all *Pseudomonas* species, the only strain with a gene homologous to *sup*6 is the opportunistic human pathogen *Ps. aeruginosa* PA7. Sup6 is 58% identical to the protein encoded by PSPA7_2111 (YP_001347484.1), which bears two cupin domains (data not shown), thus structurally categorised in the bicupins group of the cupin protein superfamily.

### Complementation of Sup^−^ Mutants

Mutants k36, R48 and W139 were complemented by a 2771 bp fragment amplified with the primers set gcd1-gcd3, which included the gene *sup*1 and a 356 bp region upstream. By promoter prediction analysis of the 356 bp region upstream, a unique promoter site was found (*P = *0.92). Complemented mutants regained the ability to inhibit radial growth of *P. ultimum* on PDA and to acidify the medium like the wild type.

In order to investigate if *sup*2, *sup*3 and *sup*4 were separately involved in biological control and acidification of PDA, several DNA fragments containing parts of the PQQ genomic locus were tested for their ability to complement mutants B91, B163, ρ26 and A150. Among these, the only one which restored the suppressive phenotype in all four mutants was a 6383 bp fragment which included five genes (*sup*2, *orf*1, *orf*2, *orf*3, *sup*3 and *sup*4) and a 504 bp region upstream of *sup*2 (Fig. 1). The 6383 bp region was PCR-amplified with primers FOR2 and pqqE2. Smaller fragments of this area and individual genes tested could not restore the sup phenotype. Complemented mutants had fully restored the ability to inhibit the radial growth of *P. ultimum* on PDA and to acidify the medium.

Complementation of sup^−^ mutants δ40 and ρ93, which bear a transposon integration in genes *sup*5 and *sup*6 respectively, could only be achieved by a 6,496 bp region which includes five genes (*sup*5, *sup*6, *orf*8, *orf*9 and *orf*7) and the 423 bp region upstream of *sup*5. Smaller DNA fragments containing only parts of this 6,496 bp region did not complement the sup- mutations in mutants δ40 and ρ93 (Fig. 2). The region that complemented the sup phenotype was PCR-amplified from *Ps. fluorescens* strain X genomic DNA with the primer set supFor-supRev.

### Analysis of the *sup5* and *sup6* Genomic Locus

The genomic locus containing genes *sup*5 and *sup*6 has not been previously implicated in the biocontrol activity of any bacterial antagonists of plant pathogens. Therefore, we studied this locus in more detail. By primer walking upstream and downstream of the transposon insertion, a total of 6,782 bp was cloned and sequenced from the region flanking these two genes in strain X. Several ORFs (open reading frames) were identified (Fig. 2), including a non-ribosomal peptide synthase (NRPS), *orf*8; a polyketide cyclase/dehydrase, *orf*9; a protein of unknown function, *orf*7; and a partial sequence of a *Emr*B/*Qac*A family drug resistance transporter, *orf*6 (data not shown). The organization of these genes in strain X is similar to the homologous loci of all fully sequenced *Pseudomonas* strains (Fig. 2) and shows high similarity with the locus attributed to mangotoxin biosynthesis by *Ps. syringae* pv. *syringae* UMAF0158 [37]. A detailed analysis of the 1,158 amino acids from the deduced product of the *orf*8 gene determined to contain a sole module [38], [39] consisting of an adenylation, a thiolation, a condensation and a reductase domain (no other domain was detected). The domains and the conserved core motifs of the NRPS encoded by *orf*8 had minimal differences with those from MgoA of the strain UMAF0158 (data not shown).

Prediction analysis of sites for putative promoters resulted in the presence of a unique promoter upstream of *sup*5 (*P = *0.96). The uniqueness of this putative promoter implies that the genes downstream might be transcribed together. Also, data resulting from the synteny search of this region revealed the existence of an operon consisting of four to five genes (Fig. 2), depending on the strain.

In this genomic locus and in the region upstream of the putative four-gene operon, most *Pseudomonas* strains examined in this study (except *Ps. aeruginosa* PA7) have a gene encoding a putative transcription factor of the GntR family. Since sequencing of the *Ps. fluorescens* strain X chromosome has not been completed for this area, it is not known whether this transcription factor exists in this strain. The potential existence of a gene encoding this transcription factor in strain X may be an indication that this factor may regulate the transcription of *sup*X in *Ps. fluorescens* strain X. Analysis of the region between the site of the unique putative promoter and the gene *sup*5, revealed a putative operator site belonging to transcriptional factors of the *Fad*R subfamily of GntR transcriptional regulators, with the sequence GAGTGGTCAGCGTTAAC. The consensus sequence for *Fad*R operators is AACTGGTCNGACCAGTT [40]. The existence of a putative operator site in the particular region, suggests that a factor of the *Fad*R subfamily might control the transcription of the genes downstream. The region containing the site of the putative promoter and the putative operator site of the transcriptional factor extends 423 bp upstream of the putative operon *sup*X (Fig. 2).

### Expression of *sup*5, *sup*6 and *orf*8 in the Wild Type and in Mutants ρ26 and k36

Quantitative gene expression analysis was performed to study the expression levels of genes *sup*5, *sup*6 and *orf*8 during growth of the wild type strain X and two selected mutants (k36, mutation in gene *sup*1 for gcd and ρ26, mutation in gene *sup*4 for PQQ) in two different liquid media (LB and PDB) during the exponential and stationary phases of growth. The media were selected because strain X inhibits *P. ultimum* radial growth on PDA but not on LA. We decided to study the expression of genes *sup*5 and *sup*6 because they are involved in the biocontrol activity of strain X, as shown by mutagenesis and complementation. We also decided to study the expression of *orf*8, a gene in the same putative operon, because it encodes a non-ribosomal peptide synthase, a class of genes responsible for the biosynthesis of antimicrobial compounds which suppress plant pathogens.

RNA from cells of the wild type and mutants was isolated from an identical stage of their growth, based on standard growth curves and a specific optical density at mid-exponential (OD_600_ = 0.6) and stationary (OD_600_ = 1) phase.

**Figure 3:**
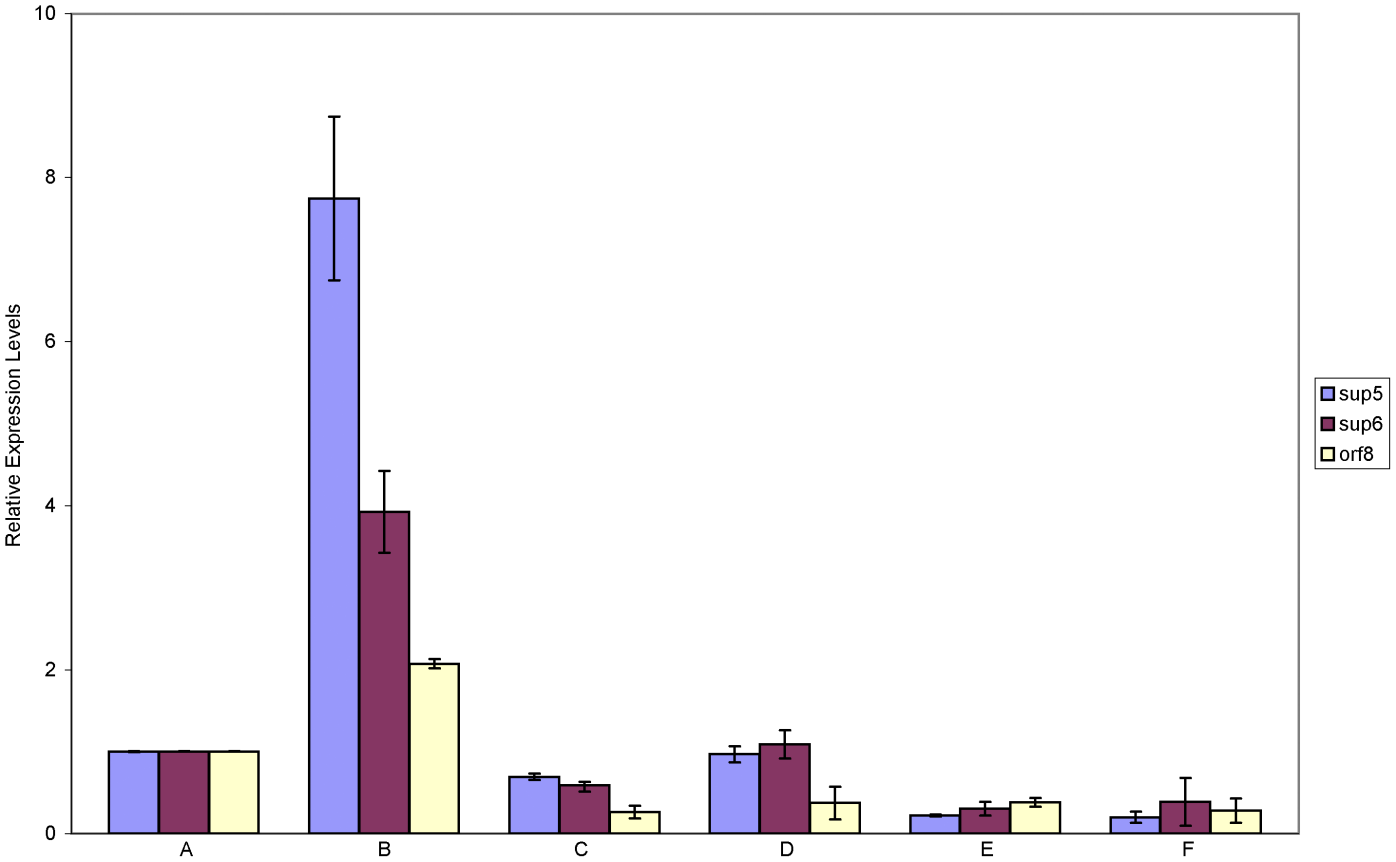
Expression levels of *sup*5, *sup*6 and *orf*8 in cells of *Ps. fluorescens* strain X and the mutants ρ26 and k36. The expression of *sup*5, *sup*6 and *orf*8 was measured using *rpo*D as the housekeeping gene standard. Results are shown as relative expression levels compared to the expression in the wild type in LB during stationary phase. (A) Expression in the wild type grown in LB, at stationary phase; (B) Expression in the wild type grown in PDB, at stationary phase; (C) Expression in mutant k36 grown in PDB, at stationary phase; (D) Expression in mutant ρ26 grown in PDB, at stationary phase; (E) Expression in the wild type grown in LB, at exponential phase; (F) Expression in the wild type strain grown in PDB, at exponential phase. For each time point, mean values of three replicates are given; the error bars represent the standard errors of the mean.

During the mid-exponential phase, transcription levels of *sup*5, *sup*6 and *orf*8 in the wild type were low and very similar in both media (Fig. 3E, 3F). At the stationary phase, transcription of *sup*X genes was low when the wild type strain was grown in LB, but much higher in PDB (Fig. 3A, 3B), in correlation with the production of an antifungal compound in PDA and the resulting reduction of radial growth. These genes exhibit the highest expression levels during the stationary phase in strain X, much like most bacterial genes responsible for the biosynthesis of secondary metabolites known to suppress plant pathogens.

Expression of *sup*5, *sup*6 and *orf*8 in the two mutants compared to the wild type was significantly decreased during the stationary phase, when bacteria were grown in PDB (Fig. 3C, 3D). Mutants ρ26 and k36 exhibited similar expression levels of genes *sup*5, *sup*6 and *orf*8 during stationary and mid-exponential phase in both nutrient media (data not shown). These results indicate that the insertional mutations in genes *gcd* and *pqq*E, which consequently result in an inactive glucose dehydrogenase, decreased the transcription of genes *sup*5, *sup*6 and *orf*8 which are responsible for the biocontrol activity of *Ps. fluorescens* strain X.

## Discussion

To elucidate the mechanism of antagonism towards the oomycete *P. ultimum*, nine mutants of the bacterial antagonist *Ps. fluorescens* strain X deficient in the *in vitro* inhibition of *P. ultimum* radial growth were isolated after mutagenesis with the mini-plasposon Tn5-RL27. Sequencing of the DNA areas flanking the transposon insertion and complementation of the mutated genes showed that three separate genomic regions are involved in the biological control traits of strain X. The biocontrol ability of *Ps. fluorescens* strain X depends on glucose dehydrogenase, its co-factor PQQ, and the proteins encoded by two additional genes (*sup*5 and *sup*6). A gene for a non-ribosomal peptide synthase (NPRS) which is located together with *sup*5 and *sup*6 in the same putative operon may also be responsible for the biocontrol activity of strain X, since NRPSs are known to synthesize antimicrobial compounds involved in the suppression of plant pathogens *in planta*.

In previous studies it has been reported that biological control traits of bacterial antagonists, such as the production of antifungal compounds, are intimately related to Gcd and its cofactor PQQ, but not in the same way for every strain. A Tn5 insertion mutant of *Ps. fluorescens* CHA0, in which the transposon had integrated in one of the PQQ biosynthetic genes (*pqq*F), showed increased production of pyoluteorin (PLT) and decreased production of 2,4-diacetylphloroglucinol (DAPG) [17]. The authors of this paper concluded that the increase in the PLT production was caused by the Tn5 integration, while the loss of DAPG production was attributed to a second spontaneous mutation that led to subsequent loss of the ability to suppress black root rot in tobacco plants and take-all in wheat. Moreover, a mutant of CHA0 carrying an in-frame deletion in the gene encoding Gcd had lost the ability to produce organic acids and to solubilise inorganic phosphate, but exhibited increased production of the antifungal metabolites DAPG and PLT. Consequently, the Δ*gcd* mutant was more effective in the biological control of *G. graminis* var. *tritici* [10]. A similar study was performed to characterize the antagonistic activity of *Rahnella aquatilis* HX2. Insertional mutations in *gcd* and *pqq*E as well as the Δ*gcd* in-frame deletion mutant were impaired in acidification of the medium and in the production of an antibacterial substance, resulting in reduced biological control of grapevine crown gall [13]. This finding shows a different effect of the same mutations in this strain. In the biocontrol strain *Serratia marcescens* W1 the genomic region of the PQQ biosynthetic genes was functionally expressed in *E. coli*. The transformed strain gained the ability to inhibit the *in vitro* radial growth of *Magnaporthe grisea* and *Cercospora citrullina* [18].

In *Ps. fluorescens* strain X, mutational inactivation of genes encoding PQQ biosynthesis proteins or Gcd resulted in loss of the *in vitro* inhibition of *P. ultimum*. This result is similar to the report on *R. aquatilis* HX2 [13] but different from the findings on *Ps. fluorescens* CHA0 [17], [10]. In accordance with the results from both HX2 and CHA0, are the results presented for the *Ps. fluorescens* strain X mutants impaired in the acidification of PDA, a glucose-containing medium (Table 1). Loss of the acidification ability in PQQ or Gcd mutants has been attributed to lack of oxidation of glucose to gluconic acid, a reaction catalysed by membrane-bound PQQ-dependant Gcd [10]. Although it has been suggested that gluconic acid is the key molecule for the biocontrol activity of strains AN5 and CHA0 [11], [10], the exact mechanism of biocontrol remains undefined in these strains.

The study of the mutants *Ps. fluorescens* δ40 and ρ93 was intriguing due to the similarity of the genes disrupted by the Tn5 insertion (*sup*6 and *sup*5) and the flanking region with the homologous genomic locus in *Ps. syringae* pv. *syringae* UMAF0158, which is involved in the biosynthesis of mangotoxin [37]. The putative product of *sup*5 was found to be similar to ABG00044.1, the product of *orf*3 in the strain UMAF0158, while *sup*6 was absent from the region involved in mangotoxin biosynthesis in the strain UMAF0158 [37]. Among all fully sequenced *Pseudomonas* strains, the only strain with a gene homologous to *sup*6 is the opportunistic human pathogen *Ps. aeruginosa* PA7. However, the putative protein encoded by *sup*6 exhibits relatively low aminoacid similarity (58%) with protein YP_001347484.1 from strain PA7, whose function is unknown.

Interesting findings were obtained from the *in silico* analysis of the genomic region in which *sup*5 and *sup*6 are localized. Synteny search concluded that these genes seem to be organized in a putative operon, designated *sup*X, together with genes *orf*7, *orf*8 (which encodes a NRPS) and *orf*9. The homologous genes in strain UMAF0158 have also been suggested to be organised in an operon [37]. From the synteny search, we also located a gene encoding a transcriptional regulator of the GntR family, upstream of this putative operon in most *Pseudomonas* strains. We do not yet have sequence data of the region upstream of *sup*X in *Ps. fluorescens* strain X, therefore we cannot confirm the presence of a GntR transcriptional regulator in this strain. However, we found a putative FadR operator site in this region, which is recognised by a transcriptional factor of the GntR family. Future work will be to sequence more of the region upstream of *sup*X and to determine the existence of a GntR-type transcriptional regulator, its operator site and the type of regulation on the transcription of the putative operon *sup*X.

The family of GntR transcriptional regulators is divided into four major subfamilies (FadR, HutC, MocR, and YtrA). DNA-binding domains, operator sequences [41], [40], as well as functions and types of regulation [41], [42] for each subfamily have been previously described. It has been demonstrated that glucose and gluconic acid hold a key role in the inactivation of regulators belonging to this superfamily [43]. Moreover, glucose inactivates *agl*3R, a GntR transcriptional repressor in *Streptomyces coelicolor*, resulting in the expression of genes involved in the ABC excretion system and antibiotic biosynthesis [44]. On the contrary, in *Serratia sp. ATCC 39006* gluconic acid inactivates the PigT transcriptional regulator, of the GntR superfamily, preventing the expression of the prodigiosin biosynthetic genes [8].

The existence of a putative FadR operator site upstream of the putative operon *sup*X is a possible indication that transcription of *sup*X might also be regulated by a factor of the GntR family. Thus, trascriptional regulation of *sup*X might be subjected to regulation by glucose, possibly through gluconic acid, the product of the catalytic action of Gcd on glucose. In order to investigate whether this hypothesis is valid, we tested the expression of three genes of the putative operon *sup*X in the wild type and in two mutants (ρ26 and k36) with deficient Gcd activity. Transcription of *sup*X correlated with the *in vitro* inhibition data of *P. ultimum* by strain X, since elevated transcription levels of *sup*5, *sup*6 and *orf*8 in the wild type were observed in PDB and not in LB. (Table 1). Expression analysis of *sup*5, *sup*6 and *orf*8 in mutants ρ26 and k36 (impaired in Gcd activity) supports the role of glucose on the antagonistic properties of strain X. Transcript levels of all three genes were significantly decreased in these mutants. The decrease was evident during the mid-exponential and stationary phase in both nutrient media tested. Finally, the expression of *sup*X was elevated during stationary phase, a typical condition during the biosynthesis of secondary metabolites. Together with the mutational and complementation analysis of *sup*X, this demonstrates that the putative operon *sup*X is responsible for the antagonistic activity of *Ps. fluorescens* strain X through the biosynthesis of an antimicrobial secondary metabolite (the chemical characterization of this metabolite is in progress).

The exact role of *sup*5 and *sup*6 in the biosynthesis of the antimicrobial metabolite is currently under investigation. Our primary hypothesis suggests that *sup*6, contributes either to the final folding of the metabolite or in the transcriptional regulation of the genes in the operon *sup*X, due to its high similarity to the functionally diverse cupin superfamily [36]. The product of *sup*5, which exhibits similarity to a heme-oxygenase-like protein, may catalyse the oxidation of the peptide chain synthesized by the NRPS encoded by *orf*8. Since the putative operon *sup*X includes a NRPS, the novel metabolite might be of peptidic nature. The results of the protease treatment of the filtrates (Table 1) do not necessarily contradict this hypothesis. Resistance of a peptidic antimicrobial compound to protease may be due to the presence of non-proteinaceous amino acids and D-amino acids, or the lack of optimum conditions for protease and proteinase cleavage (pH, final concentration of the enzyme or the antimicrobial molecule, denaturing buffer).

The results of this study demonstrated that the putative operon *sup*X is related for the first time with the biological control of a soil-borne phytopathogen by a *Ps. fluorescens* strain. Genes with some homology to those in *sup*X exist in other pseudomonads as well, including known strains with biocontrol activity, as well as plant and human pathogens. However, these genes have never been related to the biocontrol of plant pathogens before, so their role in strain X is a novel finding. Ongoing and future work will focus on the characterization of the antimicrobial compound(s) produced by strain X, the regulation of the biosynthesis of this compound by glucose and the exact role of the genes in *sup*X in the biosynthesis of this antimicrobial metabolite.

## Experimental Procedures

### Bacterial Strains, Plasmids, and Mutagenesis

All bacterial strains and plasmids used in this study are listed in Table 2. *Ps. fluorescens* strain X was grown at 28°C, and *E. coli* strains was grown at 37°C. All strains were routinely grown on LB agar, unless otherwise stated. A spontaneous derivative of *Ps. fluorescens* strain X resistant to rifampicin was used for transposon mutagenesis. This derivative had identical biochemical, growth and biocontrol characteristics with the wild type (data not shown). Transposon mutagenesis of *Ps. fluorescens* strain Xrif was carried out by triparental mating using the mini plasposon Tn-RL27, as described previously [45] and *E. coli* HB101 carrying plasmid pRK2013 [46] as the helper. This mini plasposon carries the origin of replication from plasmid R6K (*ori*R6K) to allow cloning of transposon insertion sites. Derivative strains were isolated after 48 h at 28°C on King’s B medium under selection of kanamycin (100 µg/ml) and rifampicin (40 µg/ml). In total, a library of *Ps. fluorescens* strain Xrif mutants containing over 12000 strains was constructed.

**Table 2:** Bacterial strains, plasmids, and oligonucleotides used in this study.

Strain, plasmid, oroligonucleotide		Characteristics or sequence (5′→3′)		Reference and/or source
***E. coli***				
DH10b	F^−^, mcrA, Δ(*mrr*-*hsd*RMS-*mcr*BC), Φ80d*lac*ZΔM15, Δ*lac*X74, *end*A1 *rec*A1, *deo*R, Δ(*ara*,*leu*)7697 *ara*D139, *gal*U, *gal*K, *nup*G, *rps*L, λ^−^		[54]	
DH5*a*/λpir	*sup E44*, Δ*lac*U169 (F80*lac*ZDM15), *rec*A1, *end*A1, *hsd*R17, *thi*-1, *gyr*A96, *rel*A1, λpir phage lysogen		[55]	
HB101	*hsd*R, recA,proA,leu-0,ara-l4 gaiK2, *lac*Yl, xyl-5, mtl-1 str-2, *thi*-1, *sup*E44		[48]	
***Ps. fluorescens***				
X		wild type		[35]
Xrif		Rif^R^ (spontaneous mutant)		This study
A150		Xrif derivative, sup3::Tn5-RL27 Km^R^, sup^−^		This study
B91		Xrif derivative, sup2::Tn5-RL27 Km^R^, sup^−^		This study
B163		Xrif derivative, sup4::Tn5-RL27 Km^R^, sup^−^		This study
ρ93		Xrif derivative,, sup5::Tn5-RL27 Km^R^, sup^−^		This study
ρ26		Xrif derivative, sup4::Tn5-RL27 Km^R^, sup^−^		This study
k36		Xrif derivative, sup1::Tn5-RL27 Km^R^, sup^−^		This study
R48		Xrif derivative, sup1::Tn5-RL27 Km^R^, sup^−^		This study
W139		Xrif derivative, sup1::Tn5-RL27 Km^R^, sup^−^		This study
δ40		Xrif derivative, sup6::Tn5-RL27 Km^R^, sup^−^		This study
**Plasmids**				
pBBRgcd1*		pBBR1MCS5/sup1		This study
pBBRgcd2		pBBR1MCS5/sup1		This study
pBBRpqqF		pBBR1MCS5/sup2		This study
pBBRpqqE		pBBR1MCS5/sup3		This study
pBBRpqqD		pBBR1MCS5/sup4		This study
pBBRpqqDE		pBBR1MCS5/sup3–4		This study
pBBRpqqFA*		pBBR1MCS5/sup2-orf1		This study
pBBRpqqFAB*		pBBR1MCS5sup2-orf1-orf2		This study
pBBRpqqABCDE		pBBR1MCS5/orf1–3,sup3–4		This study
pBBRpqqF-E*		pBBR1MCS5/sup2–4		This study
pBBRsup5–6		pBBR1MCS5/sup5–6		This study
pBBRsupA		pBBR1MCS5/sup6-orf7-orf8-orf9		This study
pBBRsupD*		pBBR1MCS5/sup5-sup6-orf7-orf8-orf9		This study
pRK2013		IncP-I, *tra*RK2+, *rep*RK2, *rep*E1 Km^R^		[46]
pRL27		vector of Tn5-RL27 (Km^R^-oriR6 K)		[45]
**Oligonucleotides**				
gcd1**		TCGggtaccTGAGCATTGCGTTCGCGTGAC; *Kpn* I		This study
gcd3		TCGtctagaCGCCAGCGTTGCTTAATCTG; *Xba*I		This study
FOR2		TTTGGgaatccTGACCACTCGATGTTCAGC; *EcoR*I		This study
pqqE2		GTACATCATCgaatccCGTTGAGGCGCTCA; *EcoR*I		This study
supFor		CAGCtctagaGGGAACTTGATGG; *Xba*I		This study
supRev		GCCTCCGCCTGCtctagaTATGTC; *Xba*I		This study
δ40α		CAGTGcGTGCGTcatatgAACTTCGAAGTG; *Nde*I		This study
δ40β		CCTTtctagaTGATACTCAGTAGTAGTGC; *Xba*I		This study
ρ93α		GATAAGGAGCGCcatatgGAAGATAAAAAG; *Nde*I		This study
rpoDf		GATTCGTCAGGCGATCAC		This study
rpoDr		AATACGGTTGAGCTTGTTGA		This study
rpoBf		ATCCGCAAGGACTTTAGC		This study
rpoBr		GGATAGCCAGAAGGTACG		This study
sup6f		ACGGTAGTTACTTCTTCAG		This study
sup6r		CTTCAAACAACAGGCATC		This study
sup5f		AAATCATCCTGGGCGAAG		This study
sup5r		CGAAGTGGCTGTAGTGAC		This study
orf8f		ACTATCCGTCGTGTCATCA		This study
orf8r		AAACATCACTCGCATCGTTA		This study

* includes the sequence of the putative predicted promoter.

** In the nucleotide sequences, restriction enzyme sites are shown in lowercase letters.

### Biochemical Characterization of *Ps. fluorescens* Strain X Mutants

The growth of *Ps. fluorescens* strain Xrif mutants and their ability to suppress fungal growth was tested with dual cultures on PDA at 23°C. After a first screening, potential mutants were further tested in three replicate plates together with the wild-type to confirm the loss of *P. ultimum* growth inhibition. Loss of suppression of fungal growth was assessed 2 days later.

Filtrates of strain X and selected mutants grown in PDB and LB 48 h at 23°C were tested for growth inhibition of *P. ultimum* as previously described [35].

Enzymatic treatment by pronase and proteinase K, of the filtrates was performed according to Arrebola *et al.* [47]. Observations of the culture pH were performed using different pH indicators [14].

### DNA Manipulation and Sequencing

DNA digestion, ligation reactions, and transformation of *E. coli* were performed according to standard protocols [48]. Genomic DNA isolation was performed using the GenElute™ Bacterial Genomic DNA Kit (Sigma-Aldrich Co. LLC., Germany). Plasmid mini-preps were done using the Qiaprep spin miniprep and midiprep kit (Qiagen GmbH, Düsseldorf, Germany). For sequence analysis of the regions flanking the miniplasposon insertions, published primers were used [45]. Automated DNA sequencing of rescue plasmids was carried out by Macrogen Inc. (Korea) and VBC-Biotech Service GmbH (Austria).

### Isolation and Characterization of Genomic Loci Carrying a Transposon Insertion

Plasmid rescue was performed to clone the genomic locus of the insertion in every mutant. Genomic DNA was digested with *BamH*I and subsequently treated with T4 DNA ligase. The ligation mix was transformed into *E. coli* DH5α/λpir, where circularized fragments containing the transposon replicate as plasmids, allowing the selection only of chromosomal fragments containing the transposon [45]. Southern blot analysis was performed (DIG High Primer DNA labelling and detection starter kit™, Roche Applied Science, Germany) in *BamH*I-digested genomic DNA from the selected mutants using the Tn-RL27 as probe to determine the uniqueness of the insertion in the genome and determine the size of the genomic fragments containing the transposon element.

### Complementation of Mutants

Complementation of the wild type phenotype was accomplished for all isolated strains. Specific primers designed for PCR amplification of the genes and loci in which the transposon had integrated, are listed in Table 2. Amplification of genomic loci from genomic DNA of the wild type, in which the insertion was localized in the sup^−^ mutants, was possible using the Expand Long Range dNTPack (Roche Diagnostics Gmbh, Germany). The broad host range vector pBBR1MCS5, carrying the gene for gentamycin resistance, was used for cloning single genes or genomic loci [49]. The plasmids that were constructed were transformed into *E. coli* DH10b and were subsequently inserted into the respective mutants by triparental conjugation.

### Transcriptional Analysis

For the transcriptional analyses, RNA was isolated from bacterial cells according to standard procedures [48], followed by DNase I (Promega) treatment. Total RNA was extracted from *Ps. fluorescens* strain Xrif and two mutant strains k36 and ρ26 grown in PDB and LB medium, at midlog and stationary phase. The concentration and purity of the RNA samples were measured by using an ND100 spectrophotometer (Nanodrop Technologies, USA) according to the manufacturer’s protocols. RT-PCR was performed by the SYBR® FAST One-Step qRT-PCR Kit Universal (Kapa Biosystems, USA). In every reaction, 50 ng of total RNA was used, according to the manufacturer’s protocol. Real-time quantitative PCR (Q-PCR) was conducted with the Mx3005P™ system from Stratagene (USA). The concentration of the primers was optimized (200 nM final concentration for the all genes analysed) according to the manufacturer’s technical data sheet. The primers used for the Q-PCR were chosen with the help of the Beacon Designer v 9.1software and are listed in Table 2. Control reactions in which cDNA synthesis was circumvented, ensured that DNA products resulted from the amplification of cDNA rather than from DNA contamination.

Normalization of the results was performed by using *rpo*D as the housekeeping gene [50]. The *rpo*D gene was used to provide an internal control cDNA that was amplified with oligonucleotides rpoDf/rpoDr (Table 2) and used to normalize the sample data. After the PCR, a melting curve was generated to confirm the amplification of a single product. The cycle in which the SYBR green fluorescence crossed a manually set cycle threshold (CT) was used to determine transcript levels. For each gene, the threshold was fixed based on the exponential segment of the PCR curve. Relative transcript levels of genes of interest were analyzed using the ‘Delta-delta method’ as described previously [51]. Q-PCR analysis was performed in three (technical) replicates on two independent RNA isolations (biological replicates).

### Bioinformatics

Database searches were performed with the BLAST 2.0 service of the National Center for Biotechnology Information (Bethesda, MD, U.S.A.). Amino acid sequences were aligned with MultAlin [52]. Synteny analysis was performed by using the BioCyc Database [53]. The putative promoter site prediction was performed with two bioinformatic applications, BPROM software (Softberry Inc., Mount Kisco, NY, U.S.A.) and NNPP 2.2 software (Berkeley Drosophila Genome Project-BDGP, Berkeley, CA, U.S.A.). Analysis of the cores and domains of NRPS, translated by *orf*8, was accomplished according to previous work [38], [39]. Primer design for PCR and RT-PCR was carried out using DNASTAR and BeaconDesign, respectively.

### Accession Numbers

The nucleotide and deduced amino acid sequences for the *sup*1 gene, encoding the PQQ-dependent Gcd in *Ps. fluorescens* strain *X*, has been deposited in the GenBank database under accession no. HQ383687. The nucleotide and deduced amino acid sequences for the *sup*2, *sup*3 and *sup*4 gene, encoding the PQQ biosynthesis proteins F, D and E, as parts of the flanking region in *Ps. fluorescens* strain X, have been deposited in the GenBank database under accession no. JQ039398. The nucleotide and deduced amino acid sequences of the genes located in the *sup*X putative operon, as well as parts flanking this region, have been deposited in the GenBank database under accession no. JQ039399.
